# Recurrent Volvulus of an Ileal Pouch Requiring Repeat Pouchopexy: A Lesson Learnt

**DOI:** 10.1155/2014/807640

**Published:** 2014-07-06

**Authors:** Pär Myrelid, Pelle Druvefors, Peter Andersson

**Affiliations:** ^1^Unit for Colorectal Surgery, Department of Surgery, County Council of Östergötland, 581 85 Linköping, Sweden; ^2^Division of Surgery, Department of Clinical and Experimental Medicine, Faculty of Health Sciences, Linköping University, 581 83 Linköping, Sweden; ^3^Unit for Acute Care Surgery and Trauma, Department of Surgery, County Council of Östergötland, 581 85 Linköping, Sweden

## Abstract

*Introduction*. Restorative surgery for ulcerative colitis with ileal pouch anal anastomosis (IPAA) is frequently accompanied by complications. Volvulus of the ileal pouch is one of the most rarely reported late complications and to our knowledge no report exists on reoperative surgery for this condition. *Case Report.* A 58-year-old woman who previously had undergone restorative proctocolectomy due to ulcerative colitis with an IPAA presented with volvulus of the pouch. She was operated with a single row pouchopexy to the presacral fascia. Two months later she returned with a recurrent volvulus. At reoperation, the pouch was found to have become completely detached from the fascia. A new pexy was made by firmly anchoring the pouch with two rows of sutures to the presacral fascia as well as with sutures to the lateral pelvic walls. At follow-up after five months she was free of symptoms. *Conclusion*. This first report ever on reoperative surgery for volvulus of a pelvic pouch indicates that a single row pouchopexy might be insufficient for preventing retwisting. Several rows seem to be needed.

## 1. Introduction

Ileal pouch anal anastomosis for ulcerative colitis is a complex surgical procedure frequently accompanied by short- and long-term complications [1]. The most common long-term complication is intestinal obstruction affecting up to 25% of patients [2]. Uncommon complications of pouch surgery include afferent loop syndrome and prolapse [3, 4]. Volvulus of the small bowel including the pouch or isolated volvulus of the pouch is rarely described [5–8].

## 2. Case Presentation

**Figure 1:**
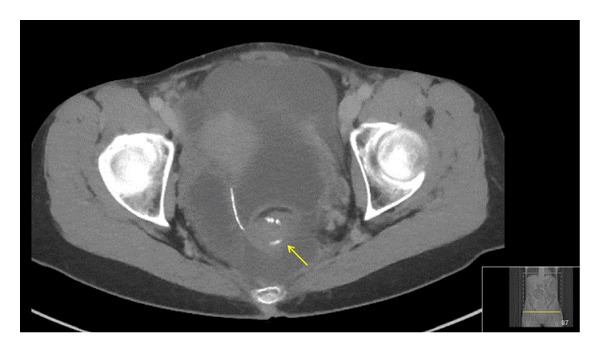
CT-scan showing volvulus of the pelvic pouch apparent by twisting of the posterior staple line 270 degrees just above the level of the ileoanal anastomosis (arrow).

**Figure 2:**
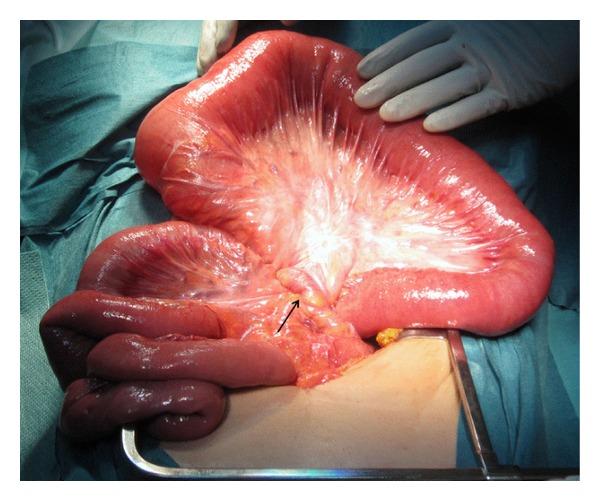
Distal ileum twisted behind the mesentery of the afferent loop (arrow).

A 58-year-old woman operated with proctocolectomy and an ileal J-pouch 11 years earlier due to ulcerative colitis complicated by colonic cancer presented at a local hospital because of abdominal pain and failure of faecal evacuation. There was a previous history of similar but short and self-resolving episodes of obstruction without clear explanation. A CT-scan aroused suspicion of twisting of the pouch along its longitudinal axis causing an obstruction at the ileoanal anastomosis. An intestinal tube was passed into the pouch for decompression which resulted in relief of symptoms lasting also the day after removal of the tube. She was therefore discharged and referred to our hospital for follow-up with pouchoscopy. However, on the same day as she had been discharged, she presented as an emergency case at our hospital with identical symptoms of obstruction and was admitted to a surgical ward after the application of a tube into the pouch for deflating. Repeated CT-scans at presentation and after application of the tube showed again volvulus of the pouch, which resolved after tube insertion as did the symptoms (Figure 1). Endoscopy of the pouch including 50 cm of the afferent loop was easily done with no signs of ischemia or obvious twisting. The following day a laparotomy was done in order to fixate the pouch to prevent further episodes of twisting. The pouch was found to be enlarged but in its regular position without twisting and not surprisingly there were no adhesions, either in the pelvis or in the abdomen, which made the pouch completely mobile. A loop of the distal ileum was also found to be twisted behind the mesentery of the afferent loop of the pouch as a remaining part of the volvulus (Figure 2). After reduction of the distal ileum a pouchopexy was made with one row of multiple interrupted nonabsorbable monofilament sutures (Prolene) to the presacral fascia as well as closure of the open space behind the mesentery of the afferent loop in the same manner. The postoperative course was uneventful and the patient was discharged.

**Figure 3:**
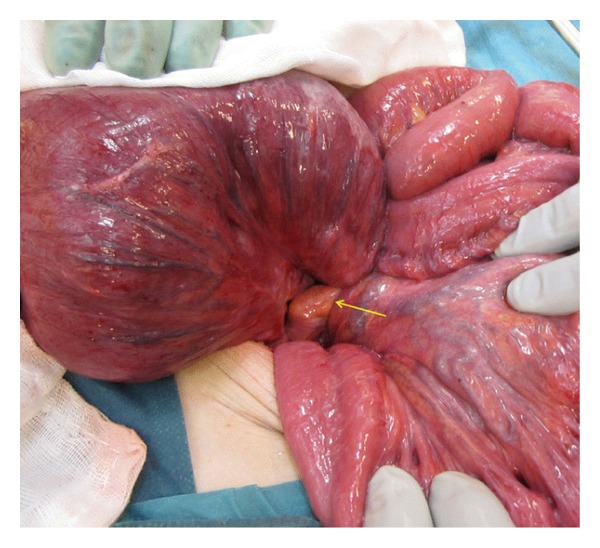
The enlarged pouch twisted around the long axis of its mesentery (arrow).

**Figure 4:**
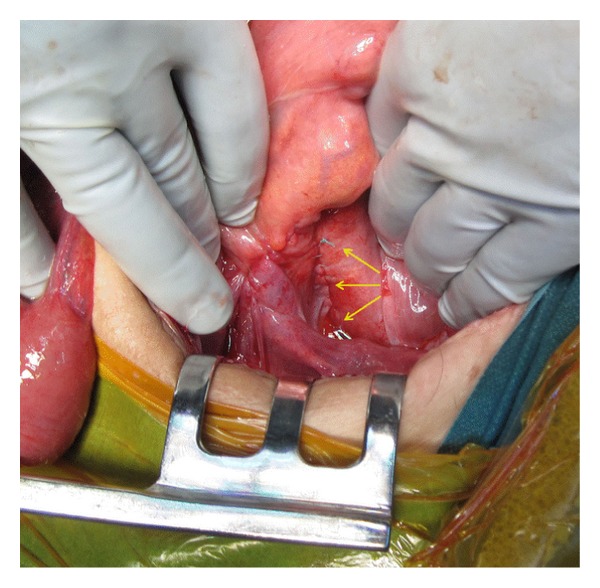
Pouchopexy by suturing the pouch to the presacral fascia on either side of the mesentery of the pouch (arrows).

Two months later the patient presented with identical symptoms and a CT-scan showed again volvulus of the pouch. Insertion of a tube on this occasion did not fully relieve symptoms, so this led to a decision to proceed to laparotomy. A volvulus of the pouch along its longitudinal axis was found intraoperatively as well as an accompanying volvulus of the small bowel behind the afferent loop similar to the previous laparotomy (Figure 3). No new adhesions had been formed since that laparotomy and all the sutures to the presacral fascia and retroperitoneum were completely detached except for one. The mildly ischemic pouch and small bowel were derotated and fixated once again to the sacral fascia and retroperitoneum but this time with two rows of continuous multifilament sutures (Ethibond) on either side of the pouch. The sutures were also extended upwards to again close the open space behind the afferent loop mesentery (Figure 4). Furthermore the upper corners of the pouch were sutured to the lateral walls of the pelvis. After an uncomplicated postoperative course, the patient was discharged. At follow-up ten months later she was doing well and was free of symptoms.

## 3. Discussion

Volvulus of a pelvic pouch is a very rare condition. Presumably our patient had been suffering from intermittent partial twisting of the pouch for several years, but making a correct diagnosis of this condition had been almost impossible. To the best of our knowledge, only three cases of true pelvic pouch volvulus have been described in the literature [9–11]. In two cases the pouch was excised due to necrosis, but in the third it was possible to retain the pouch and, as in our case, to carry out pouchopexy but to do so anteriorly to the bowel wall in contrast with our procedure. A prerequisite for volvulus to occur is the absence of adhesions within the lower pelvis. As a consequence of the patient's inability to form fibrous adhesions around the pouch, pexy of the pouch in case of twisting has to be firmly done. Our initial ignorance of this fact as well as our unawareness of the considerable weight of a full and movable pouch resulted in creating too weak an anchoring of the pouch to the presacral fascia, with a recurrence shortly after. Luckily the patient did not develop a devastating ischemia in the pouch when the volvulus recurred but could instead be treated with renewed pouchopexy by means of double rows of nonabsorbable multifilament sutures that we expect to endure in the long run. The multifilament component was chosen in order to induce some fibrosis and subsequent adhesions at the site of the anchoring. The patient has been doing well far after the second operation. This is, as far as we know, the first time that a repeated surgery for a recurrent volvulus of a pelvic pouch has been described.

## 4. Conclusion

The case in our opinion is of general interest due to the fact that intermittent volvulus should be borne in mind as a possible, if rare, cause of episodic obstruction in patients with a pelvic pouch. Furthermore, if a volvulus of a pelvic pouch is found at emergency laparotomy, a single row pouchopexy presumably is not sufficient to prevent retwisting. Therefore, we advocate firm anchoring to the sacral fascia using at least two rows of nonabsorbable sutures.
